# A Rare Case of *TP63*-Associated Lymphopenia Revealed by Newborn Screening Using TREC

**DOI:** 10.3390/ijms251910844

**Published:** 2024-10-09

**Authors:** Andrey Marakhonov, Elena Serebryakova, Anna Mukhina, Anastasia Vechkasova, Nikolai Prokhorov, Irina Efimova, Natalia Balinova, Anastasia Lobenskaya, Tatyana Vasilyeva, Victoria Zabnenkova, Oxana Ryzhkova, Yulia Rodina, Dmitry Pershin, Nadezhda Soloveva, Anna Fomenko, Djamila Saydaeva, Aset Ibisheva, Taisiya Irbaieva, Alexander Koroteev, Rena Zinchenko, Sergey Voronin, Anna Shcherbina, Sergey Kutsev

**Affiliations:** 1 Research Centre for Medical Genetics, 115522 Moscow, Russia; efimova.geneticist@gmail.com (I.E.); balinovs@mail.ru (N.B.); vasilyeva_debrie@mail.ru (T.V.); zabnenkova.vv@gmail.com (V.Z.); ryzhkova@dnalab.ru (O.R.); renazinchenko@mail.ru (R.Z.); voronin.sv@med-gen.ru (S.V.); kutsev@mail.ru (S.K.); 2 Saint-Petersburg State Medical Diagnostic Center (Medical Genetic Center), 194044 Saint-Petersburg, Russia; el.a.serebryakova@mail.ru (E.S.); vechkasova.nastia@mail.ru (A.V.); lobenskaya@mail.ru (A.L.); alexkoroteev@mail.ru (A.K.); 3 Dmitry Rogachev National Medical Research Center of Pediatric Hematology, Oncology and Immunology, 117198 Moscow, Russia; ffmanya@yandex.ru (A.M.); rodina.julija@rambler.ru (Y.R.); dimprsh@icloud.com (D.P.); shcher26@hotmail.com (A.S.); 4 Department of Molecular and Cellular Biochemistry, Indiana University, Bloomington, IN 47405, USA; niprokho@iu.edu; 5 Department of Neonatal and Infantile Pathology, Saint-Petersburg State Pediatric Medical University, 194100 Saint-Petersburg, Russia; nadia82@bk.ru (N.S.); fomenkoanna8515@gmail.com (A.F.); 6 State Budgetary Institution “Maternity Hospital” of the Ministry of Healthcare of the Chechen Republic, 364017 Grozny, Russia; saydaeva78@mail.ru (D.S.); ibisheva18@mail.ru (A.I.); 7 Department of Maternity and Childhood, Ministry of Healthcare of the Chechen Republic, 364061 Grozny, Russia; irbaieva_mz@mail.ru

**Keywords:** newborn screening, TREC, KREC, lymphopenia, ectrodactyly, ectodermal dysplasia, cleft-lip/palate, *TP63*

## Abstract

The expanded newborn screening (NBS) program in the Russian Federation was initiated in 2023, among which severe combined immunodeficiency (SCID) is screened using TREC/KREC assays. Here, we report a rare case of a *TP63*-associated disease identified through this NBS program. Dried blood spots from newborns were initially screened for TREC/KREC levels, and those with values below the cut-off underwent confirmatory testing and further genetic analysis, including whole-exome sequencing (WES). A male newborn was identified with significantly reduced TREC values, indicative of T cell lymphopenia. Genetic analysis revealed a heterozygous NM_003722.5:c.1027C>T variant in *TP63*, leading to the p.(Arg343Trp) substitution within the DNA binding domain. This mutation has been previously associated with Ectrodactyly–Ectodermal Dysplasia–Cleft lip/palate syndrome (EEC) syndrome and shown to reduce the transactivation activity of TP63 in a dominant-negative manner. This case represents one of the few instances of immune system involvement in a patient with a *TP63* mutation, highlighting the need for further investigation into the immunological aspects of *TP63*-associated disorders. Our findings suggest that comprehensive immunological evaluation should be considered for patients with *TP63* mutations to better understand and manage potential immune dysfunctions.

## 1. Introduction

Primary immunodeficiencies (PIDs), also referred to as inborn errors of immunity (IEIs), are a diverse group of genetically inherited disorders characterized by abnormalities in various components of the immune system [1].

Newborn screening (NBS) for severe combined immune deficiency (SCID), which involves measuring T-cell receptor excision circles (TRECs) in dried blood spots on filter paper cards, is successfully conducted in many countries worldwide [2].

In addition to SCID, other forms of PID, especially congenital thymic defects (22q11.2 deletion syndrome, CHARGE syndrome, FOXN1 deficiency, TBX1 deficiency, etc.) can also be identified through newborn screening [3,4]. These findings highlight the importance of broad and comprehensive screening strategies to detect a wide range of immunodeficiency disorders early on, thereby facilitating timely interventions to improve clinical outcomes.

TP63 is a critical transcription factor involved in regulating the development of ectodermal tissues, limbs, and orofacial structures [5,6]. Pathogenic variants in the *TP63* gene are linked to a spectrum of syndromes, including Ectrodactyly, Ectodermal Dysplasia, and Cleft Lip/Palate Syndrome 3 (EEC3); Ankyloblepharon-Ectodermal Defects-Clefting (AEC) Syndrome; Split-Hand/Foot Malformation 4; Orofacial Cleft 8; and others [7]. These syndromes predominantly affect ectodermal structures and limb formation. However, immune system abnormalities have been rarely documented in patients with *TP63*-associated disorders [8,9].

Since 2023, an expanded NBS program has been initiated in the Russian Federation [10]. The current protocol includes screening for severe T cell (measuring TREC) and B cell deficiencies (measuring kappa-chain excision circles, KREC). Here, we report a rare case of *TP63*-associated disease identified as a result of NBS for IEI using TREC and KREC detection.

## 2. Results

The male patient was the sixth child in a non-consanguineous family without significant family history; all siblings were reported to be healthy. He was born with a birth weight of 3170 g and length of 50 cm, at 38 weeks 5 days to a 37-year-old mother via emergency C-section for placental abruption. Ultrasound screening at 33 weeks of gestation revealed bilateral cheilognathopalatoschisis and bilateral megaureter with hydronephrosis.

**Figure 1:**
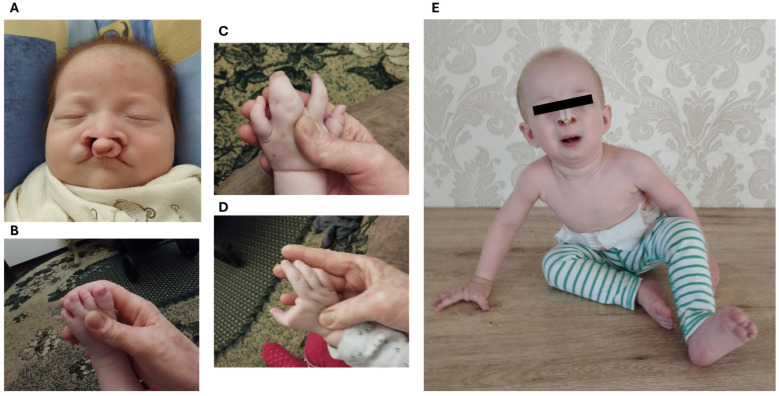
Clinical picture of the patient. (**A**)—facial view of the patient in the perinatal period demonstrating bilateral cheilognathopalatoschisis. (**B**)—syndactyly of 3–4 toes on the left foot. (**C**)—complete syndactyly of 3–4 fingers on the left palm. (**D**)—syndactyly of rudimentary 2nd finger with full 1st finger of the right palm (split hand). (**E**)—view of the patient at the age of 1-year-old demonstrating condition after reconstructive surgery of facial structures, split right hand and left foot.

Birth Apgar scores were normal (7/8). The patient had bilateral cheilognathopalatoschisis with complete cleft lip/cleft palate, incomplete atresia of the nasal septum, lacrimal ductus atresia, and complete syndactyly of 3–4 fingers on the left palm, syndactyly of rudimentary 2nd finger with full 1st finger of the right palm (split hand), and complete syndactyly of 3–4 toes of the left. The skin on the back was peeling, and sparse hair was also noted (Figure 1).

On the second day of life, the dry blood spot sample was referred to the Center of the expanded NBS at the Medical Genetic Center (Diagnostic), Saint-Petersburg, where a decrease in TREC level down to 35 copies/10^5^ cells was determined with a normal level of KREC (450 copies/10^5^ cells). At the age of 8 days, second-tier PCR resulted in a TREC level of 175 copies/10^5^ cells with a normal level of KREC (9422 copies/10^5^ cells). On the 19th day of life, flow cytometry assay demonstrated slightly decreased T cell counts (1.928 × 10^9^/L) with a decrease in CD4+ naïve populations (CD3+CD4+CD45RA+CD197+ 66.2%; CD3+CD8+CD45RA+CD197+ 91.5%), and normal B-cell counts (1.787 × 10^9^/L) (Table 1). Taking into account severe congenital malformations, the patient was referred for genetic testing.

**Figure 2:**
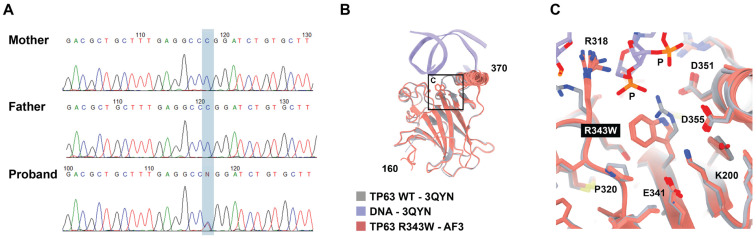
Genetic examination of the patient: (**A**) Sanger sequencing of the nuclear family revealed NM_003722.5(TP63):c.1027C>T, p.(Arg343Trp), variant occurred de novo (highlighted in blue background). (**B**) Modeling of the substitution in the paired domain of the TP63 protein. The five Alphafold 3 computed structure models (CSMs) of the R343W variant from the same in silico experiment are superimposed with the previously experimentally determined crystal structure of TP63 (PDB accession number 3qyn). The CSMs are colored pink, and the crystal structure of WT TP63 with DNA is gray and purple. The section enclosed within the square frame is further magnified in (**C**). (**C**) Zoomed-in view of the same superposition in the proximity to the mutation showing the disappearance of a salt bridge between the DNAbinding domain of the R343W variant and the sugar–phosphate backbone.

Whole-exome sequencing revealed a previously reported [11,12] single nucleotide variant NM_003722.5:c.1027C>T in a *TP63* gene in a heterozygous state. It is worth noting that no alternative underlying causes for the observed T-cell lymphopenia were identified in the patient, suggesting that the immune deficiency is most likely associated with the *TP63* variant. This variant results in a missense substitution of the highly conservative amino acid residue p.(Arg343Trp) within the DNAbinding domain of the TP63 protein. This variant is absent in the gnomAD v2.1.1 exomes and genomes database [13] as well as in the gnomAD v3.1.2 exomes and genomes database [14]. Multiple bioinformatic tools predict the variant to be deleterious. Taking into account these data, the variant is classified to be likely pathogenic with the criteria PM2, PS1, and PP5 according to the ACMG recommendations [15]. We next assessed the segregation of the variant in the family and revealed that it occurred de novo (Figure 2A).

The functional consequences of the identified missense substitution were further analyzed using several bioinformatic tools. DynaMut2 [16] predicted a stability change ΔΔG^Stability^ of −0.767 kcal/mol, suggesting that the variant may have a destabilizing effect on the protein. This potential mechanism of action is further supported by 3D visualization, which shows that the positively charged Arg-343 residue has direct contact with a sugar–phosphate backbone of DNA, and the Arg-to-Trp non-conservative substitution abolishes this interaction (Figure 2B,C).

According to the genetic testing and a clinical picture, the proband has a diagnosis of EEC3 syndrome (ectrodactyly, ectodermal dysplasia, and cleft lip/palate syndrome-3) (OMIM #604292).

A 1-year follow-up of the patient revealed that lymphocyte subpopulations and serum immunoglobulin levels remained within normal ranges (Table 1). The patient demonstrated normal developmental progress, reaching key milestones on time: holding his head at 1.5 months, sitting at 6 months, and walking at 13 months of age. During this period, he underwent multiple reconstructive surgeries for urinary tract malformations as well as cleft lip and cleft palate repair. He suffered from recurrent urinary tract infections, which required multiple courses of antimicrobials. Noncomplicated episodes of otitis media and bronchitis were also reported.

## 3. Discussion

TP63 (Tumor Protein p63) is a critical member of the p53 family of transcription factors, which includes p53 and p73 [17]. This protein plays a multifaceted role in a variety of cellular processes. TP63 plays a critical role in the regulation of development, differentiation, and maintenance of epithelial tissues. TP63 can also induce apoptosis, similar to p53, particularly in response to DNA damage and cellular stress [18]. While TP63 can function as a tumor suppressor, its overexpression or mutations can also be associated with certain cancers. The balance of TP63 activity is crucial for preventing tumorigenesis [19]. As a transcription factor, TP63 regulates the expression of various target genes involved in cell adhesion, differentiation, proliferation, and apoptosis [20]. TP63 is important for the maintenance of stem cell properties in basal epithelial cells [21]. TP63 is essential for the development and maintenance of stratified epithelial tissues, such as the skin, limbs, and craniofacial structures. It is highly expressed in basal cells of epithelial tissues, where it promotes the proliferation and differentiation necessary for tissue formation and regeneration [22].

Heterozygous mutations in *TP63* are linked to several congenital syndromes, such as Ectrodactyly–Ectodermal Dysplasia–Cleft lip/palate (EEC) syndrome [11], Ankyloblepharon-Ectodermal Dysplasia-Clefting (AEC) syndrome, and others, which affect the development of epithelial tissues and structures [6]. The diverse range of conditions linked to *TP63* mutations is considered part of a spectrum of multiple-congenital-anomaly syndromes that impact limb and ectodermal structures. Research has established a clear genotype–phenotype correlation, with specific mutations clustering in distinct domains of the TP63 protein. Mutations in the DNA binding domain (DBD) are primarily associated with EEC syndrome, whereas mutations in the SAM (sterile-alpha motif) domain are linked to AEC syndrome [6].

The NM_003722.5:c.1027C>T variant found in our patient affects the ultraconservative amino acid residue p.(Arg343Trp) in the DNA binding domain (DBD) of the TP63 protein. This variant has been previously reported in multiple patients with Ectrodactyly–Ectodermal Dysplasia–Cleft lip/palate (EEC) syndrome [23]. The p.(Arg343Trp) mutation has been shown to maintain stable expression levels but exhibited a marked reduction in the transactivation activity of TP63 in a dominant-negative manner [24]. These functional data strongly support the hypothesis that Arg343Trp substitution should destabilize the TP63-DNA contacts.

The role of TP63 in immune system development and its involvement in the immune presentation of *TP63*-associated diseases is not well understood. TP63 is expressed as a marker of epithelial cells in the thymus [25], which is the primary lymphoid organ responsible for the generation and maturation of T cells. Thymic epithelial cells (TECs) constitute the majority of the thymic stroma [26] acting as the essential cells in the coordination of T cell development [27]. Various genes involved in thymus development are implicated in the pathogenesis of athymic syndromes [28].

One of two isoforms of TP63 (N-terminal truncated/Delta—ΔNp63 isoform) is expressed in TEC, maintaining the proliferation of epithelial stem cells [29]. Fibroblast growth factor receptor 2-IIIb (FgfR2-IIIb), essential for TEC proliferation, and Notch ligand (Jag), which supports T cell development, are the only known p63 targets to date [30]. Loss of p63 results in thymic hypoplasia with a reduction in TEC and thymocyte numbers and a decrease in TEC proliferative colonies numbers [30,31]. Morphological markers of TEC and, furthermore, TEC commitment for T cell development demonstrate that p63 is not required for functional maturation of thymic epithelial cells. Developing thymocytes progress normally expressing surface markers CD4, CD8, and TCR independently to p63 [27,31]. Indeed, our patient exhibits only a mild decrease in CD4+ and CD8+ lymphocyte counts, both at the time of initial screening and during the one-year follow-up. It is important to note that the recurrent infections observed in our patient were primarily related to complications arising from urinary tract malformations and multiple surgeries. These infectious episodes were not characterized by a prolonged course and displayed typical severity, suggesting that they are unlikely to be directly associated with immune dysfunction. The changes observed in our patient appear to be clinically insignificant at this time. Nevertheless, they underscore the need for continued monitoring and follow-up to ensure timely identification and management of any potential deterioration in the future. There have been reports of immune system involvement in patients with Ectrodactyly–Ectodermal Dysplasia–Cleft lip/palate (EEC) syndrome, a condition associated with TP63 mutations [8,9]. Four infants had TREC levels below cut-off values [9,12] with mild T-lymphopenia in all of them and decreased CD8+ counts in one [9]. Improvement in T cell counts with sustained normal T cell function was observed in all of them [8]. Nevertheless, two infants required immunoglobulin replacement therapy until the recovery of T cell counts.

**Figure 3:**
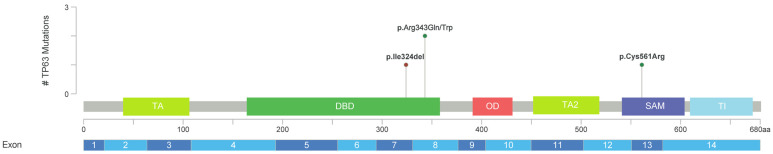
Locations of TP63 variants identified in patients with immune system abnormalities. The domain structure is illustrated based on the TAp63α isoform [34]: TA—transactivation domain, DBD —DNA binding domain, OD—oligomerization domain, SAM—sterile-alpha motif domain, TI—transactivation inhibitory domain. Figures were created and modified by using the MutationMapper (https://www.cbioportal.org/mutation_mapper, accessed on 9 September 2024) [35].

Notably, the twins described by Wenger et al. 2018 bear the other pathogenic missense variant NM_003722.5:c.1028G>A, p.(Arg343Gln), which also affects the same amino acid residue, Arg-343, that is substituted in our patient. Both variants are located within the DNAbinding domain (DBD) of the TP63 protein [12]. Similar to the p.(Arg343Trp) mutation, the p.(Arg343Gln) variant has also been demonstrated to abolish p63 activity [32]. Additionally, another reported patient carries a trinucleotide pathogenic deletion, NM_003722.5:c.970_972delATT, resulting in the in-frame deletion of one amino acid residue, p.(Ile324del), also within the DBD (Figure 3) [8]. Furthermore, a fourth premature patient with a phenotype overlapping between EEC and AEC syndromes was found to have a pathogenic missense variant, NM_003722.4:c.1681T>C, p.(Cys561Arg), affecting the SAM domain of TP63 [9], despite the fact that variants in the SAM domain are typically associated with AEC syndrome [6]. It should be noted that functional studies for the two latter variants, p.(Ile324del) and p.(Cys561Arg), have not been performed. This comparative analysis suggests that while the majority of variants linked to immune abnormalities are localized within the DBD of TP63, mutations affecting other regions, such as the SAM domain (often linked to different phenotypes), may also potentially result in immune system involvement.

Akahoshi et al. described a Japanese girl with genetically confirmed EEC syndrome and diffuse large B-cell non-Hodgkin lymphoma associated with decreased levels of IgG, IgA, and IgM diagnosed at the age of 16 years old [33]. Our patient displays normal levels of immunoglobulins at the age of 12 months but requires immunological follow-up concerning neoplastic processes.

These observations suggest that the immune status of patients with *TP63*-associated disorders is understudied and warrants further investigation. Future research could include both in vitro assays, focusing on the analysis of lymphocyte development in the context of the TP63 variants, as well as in vivo models to evaluate the physiological effects of this variant on the immune system and overall health. These approaches will be essential in elucidating the mechanistic role of TP63 in immune function and could ultimately inform clinical management strategies for affected patients.

## 4. Materials and Methods

The expanded NBS program was conducted as previously described [10]. Written parental informed consent was obtained for all newborns enrolled in the NBS program. Initially, dried blood spot (DBS) cards were collected on the second day for term newborns and on the seventh day for preterm newborns. These samples were then delivered to 10 Centers of Expanded NBS, including the Medical Genetic Center (Diagnostic) in Saint-Petersburg. At these centers, the samples were analyzed by quantitative PCR (qPCR) for TREC, KREC, and the homozygous deletion of exon 7 of the *SMN1* gene using the TK-SMA assay (ABV-Test, Moscow, Russia) as described elsewhere. The cut-off values for TREC/KREC were determined to be 100 copies/10^5^ cells.

Further, newborns whose first-tier PCR results fell below the cut-off were referred to the Research Centre for Medical Genetics for second-tier PCR using the Eonis™ SCID-SMA kit (Wallac Oy, Turku, Finland) on Applied Biosystems QuantStudio 5 Dx instruments (Thermo Fisher Scientific, Waltham, MA, USA) as previously described [36]. TREC and KREC concentrations (copies/10^5^ cells) were calculated by the Eonis™ Analysis software IVD v.1.1 (Wallac Oy, Turku, Finland). If a decrease in TREC and/or KREC levels was confirmed in the dried blood spots (DBSs), or in case of the presence of a clinical picture, the newborn underwent immunological examination and DNA diagnostics, including whole-exome sequencing (WES) [37].

Whole-exome sequencing (WES) was performed on genomic DNA samples from the patients on NextSeq 500 (Illumina, San Diego, CA, USA) in 2 × 150 bp paired-end mode. The bioinformatics pipeline of NGS data analysis was described previously [38]. In brief, it included quality control of raw reads (FastQC tool v0.11.5), followed by read mapping to the hg19 human genome assembly (minimap2 v2.24-r1122), sorting of the alignments, and marking duplicates (Picard Toolkit v2.18.14). Base recalibration and variant calling were performed with GATK3.8. Variant annotation was carried out using ANNOVAR (v2018Apr16). CNV and SV analysis was performed using the Manta tool (v1.6.0). Causative variants discovered by WES were validated by Sanger sequencing in the patient and parents. Variants were named according to the hg19 human genome assembly and the NM_003722.5 transcript variant as a reference.

All biological samples were deposited into the Moscow Branch of the Biobank “All-Russian Collection of Biological Samples of Hereditary Diseases”.

Alphafold 3 was used to generate computed structure models (CSMs) of the R343W variant of TP63 [39]. CSMs were visualized with UCSF ChimeraX v1.7 [40].

## 5. Conclusions

The identification of a patient with a *TP63* mutation through the NBS using TREC/KREC exhibiting T-cell lineage involvement underscores the potential significance of TP63 in immune system development. This case highlights the necessity for comprehensive immunological assessments in patients with *TP63*-associated disorders. Given the limited understanding of TP63’s role in the immune system, further research is needed to elucidate the mechanisms by which *TP63* mutations impact immune function. Detailed investigations could reveal novel insights into the pathophysiology of *TP63*-related syndromes and lead to improved diagnostic and therapeutic strategies for affected individuals.

## Figures and Tables

**Table 1:** Immunological examination of the patient.

Parameter	19 Days of Life *	12 Months of Life	Normal Range
T cells (CD3+), 10^9^/L	1.92	2.01	3.3–6.5 (58–71%)
Th (CD4+), 10^9^/L (% of T cells)	1.22 (63.4%)	1.15 (57.2%)	1.9–4.4 (56–79%)
Th naïve (CD45RA+CD197+), cells/µL (% of CD4+)	809 (66.2%)	773.52 (67.2%)	1442–3731 (72–86%)
T-central memory, cells/µL (% of CD4+)	284 (23.3%)	115(10%)	151–448 (4.5–15.1%)
Effector memory, cells/µL (% of CD4+)	105 (8.6%)	199 (17.3%)	126–399 (4.2–13.9)
TEMRA, cells/µL (% of CD4+)	24 (2%)	64 (5.6%)	36–225 (1.3–6.9%)
Tc (CD8+), 10^9^/L (% of T cells)	0.66 (34%)	0.73 (36.1%)	0.7–1.9 (18–39%)
Tc naïve (CD45RA+CD197+), cells/µL (% of CD8+ cells)	600 (91.5%)	352 (48.5%)	455–1515 (34–88%)
T-central memory, cells/µL (% of CD8+)	37 (5.6%)	18 (2.6%)	6–85 (0.4–8.0%)
Effector memory, cells/µL (% of CD8+)	11 (1.7%)	245 (33%)	50–395 (3.4–24.4%)
TEMRA, cells/µL (% of CD8+)	9 (1.3%)	109 (15.1%)	69–736 (6.1–39.0%)
B cells (CD19+), 10^9^/L (% of Lymphocytes)	1.78 (40%)	2.57 (45%)	1–3 (17–36%)
B naïve (CD19+IgD+IgM+CD27−), cells/µL (% of B cells)	n/a	2360 (91.9%)	290–1637 (62–83%)
Transitional B cells (CD19+IgM+CD38+), cells/µL (% of B cells)	n/a	41 (1.6%)	32–235 (3.4–11.9%)
Natural effector B cells (IgD+IgM+CD27+), cells/µL (% of B cells)	n/a	67 (2.6%)	15–242 (2.7–14.6%)
IgM-only B cells (CD27+IgM+IgD−), cells/µL (% of B cells)	n/a	5 (0.2%)	2–28 (0.2–2.0%)
Switched B memory cells (IgD−IgM−CD27+), cells/µL (% of B cells)	n/a	36 (1.4%)	5–83 (0.5–5.1%)
Plasmablasts (CD19+IgD−IgM−CD27+CD38^high^), cells/µL (% of B cells)	n/a	2.6 (0.1%)	1–28.5 (0.07–3.17%)
Plasma B cells (CD19+IgD−IgM−CD27+CD38^high^CD138+), cells/µL (% of B cells)	n/a	0 (0%)	–
CD21^low^CD38^low^ B cells, cells/µL (% of B cells)	n/a	72 (2.8%)	7–75 (1.1–5.0%)
NK cells (CD3−CD16+CD56+), 10^9^/L (% of Lymphocytes)	0.7 (15.9%)	0.9 (16.1%)	0.2–1.3 (3–16%)
CD56+high NK, cell/µL (% of NK-cells)	100 (15.6%)	70 (14%)	10–160 (2.6–15.3%)
T-NK-cells CD3+CD56+, cell/µL (% of Lymphocytes)	0	6 (0.1%)	5–30 (0.05–0.39%)
IgG, g/L	n/a	7.02	4.75–12.10
IgM, g/L	n/a	0.87	0.41–1.83
IgA, g/L	n/a	0.55	0.21–2.91

* n/a—not assessed.

## Data Availability

The datasets used and/or analyzed during the current study are available from the corresponding author upon reasonable request.
